# Supplementary material for: Causal Relationship between Aging and Anorexia Nervosa: A White-Matter-Microstructure-Mediated Mendelian Randomization Analysis
Source: Biomedicines. 2024 Aug 16;12(8):1874. doi: 10.3390/biomedicines12081874 (PMC11351342; doi:10.3390/biomedicines12081874)
Supplement: Supplementary file 1 [file biomedicines-12-01874-s001.zip › Supplementary Figure/Figure S4/Singe SNP plot of MR analysis for TL to ubm-b-1715.pdf]

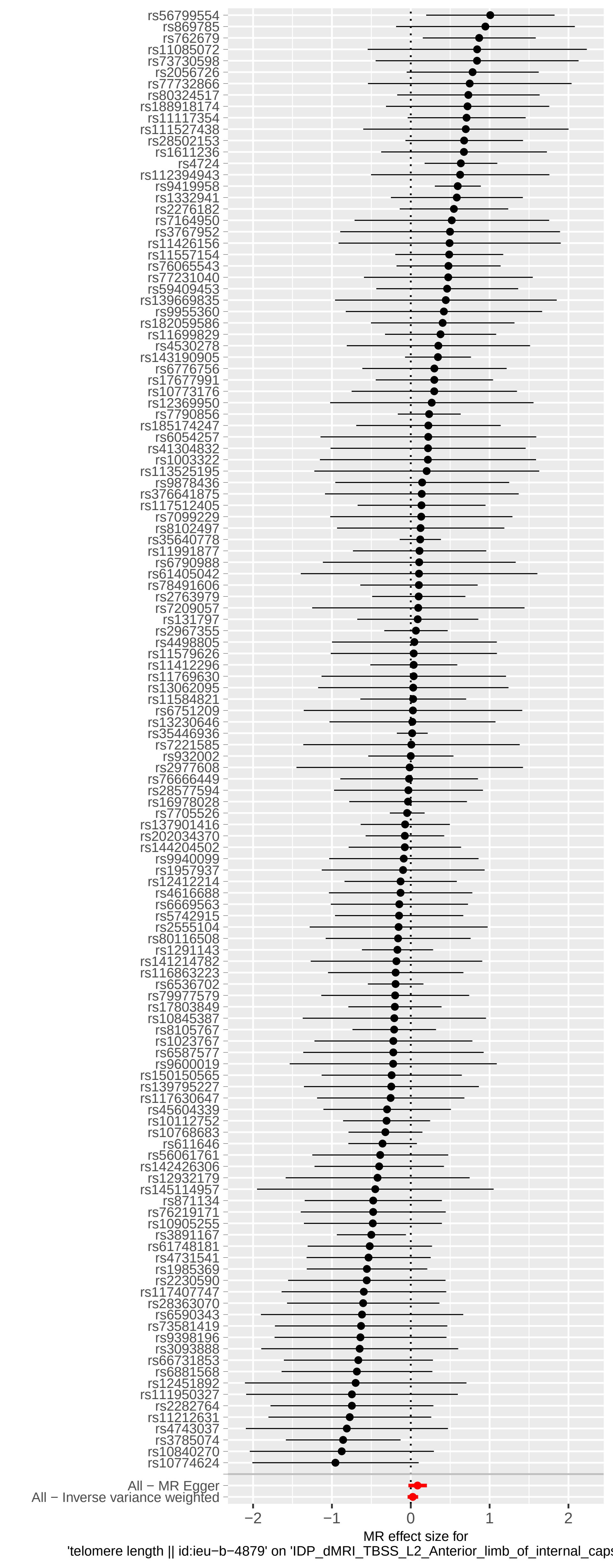
rs56799554
rs869785
rs762679
rs11085072
rs73730598
rs2056726
rs77732866
rs80324517
rs188918174
rs11117354
rs111527438
rs28502153
rs1611236
rs4724
rs112394943
rs9419958
rs1332941
rs2276182
rs7164950
rs3767952
rs11426156
rs11557154
rs76065543
rs77231040
rs59409453
rs139669835
rs9955360
rs182059586
rs11699829
rs4530278
rs143190905
rs6776756
rs17677991
rs10773176
rs12369950
rs7790856
rs185174247
rs6054257
rs41304832
rs1003322
rs113525195
rs9878436
rs376641875
rs117512405
rs7099229
rs8102497
rs35640778
rs11991877
rs6790988
rs61405042
rs78491606
rs2763979
rs7209057
rs131797
rs2967355
rs4498805
rs11579626
rs11412296
rs11769630
rs13062095
rs11584821
rs6751209
rs13230646
rs35446936
rs7221585
rs932002
rs2977608
rs76666449
rs28577594
rs16978028
rs7705526
rs137901416
rs202034370
rs144204502
rs9940099
rs1957937
rs12412214
rs4616688
rs6669563
rs5742915
rs2555104
rs80116508
rs1291143
rs141214782
rs116863223
rs6536702
rs79977579
rs17803849
rs10845387
rs8105767
rs1023767
rs6587577
rs9600019
rs150150565
rs139795227
rs117630647
rs45604339
rs10112752
rs10768683
rs611646
rs56061761
rs142426306
rs12932179
rs145114957
rs871134
rs76219171
rs10905255
rs3891167
rs61748181
rs4731541
rs1985369
rs2230590
rs117407747
rs28363070
rs6590343
rs73581419
rs9398196
rs3093888
rs66731853
rs6881568
rs12451892
rs111950327
rs2282764
rs11212631
rs4743037
rs3785074
rs10840270
rs10774624
All − MR Egger
All − Inverse variance weighted
−2
−1
0
1
2
MR effect size for
'telomere length || id:ieu−b−4879' on 'IDP_dMRI_TBSS_L2_Anterior_limb_of_internal_caps
